# User-relevant factors influencing the prosthesis use of persons with a transfemoral amputation or knee-disarticulation: A meta-synthesis of qualitative literature and focus group results

**DOI:** 10.1371/journal.pone.0276874

**Published:** 2023-01-17

**Authors:** Charlotte E. Bosman, Corry K. van der Sluis, Jan H. B. Geertzen, Nienke Kerver, Aline H. Vrieling

**Affiliations:** Department of Rehabilitation Medicine, University of Groningen, University Medical Center Groningen, Groningen, The Netherlands; Brunel University London, UNITED KINGDOM

## Abstract

**Objective:**

Persons with a transfemoral amputation or knee-disarticulation are heavily reliant on an adequate set of components for their prosthesis. To improve the process of adjusting the specific prosthetic properties to the expectations of the prosthesis users, it is of importance to first identify which factors have an influence on prosthesis use. Therefore, we aimed to identify factors that influence prosthesis use in adults with a transfemoral amputation or knee-disarticulation.

**Methods:**

A qualitative meta-synthesis was conducted by searching five databases (last update January 20^th^ 2022). Studies were considered eligible if they contained qualitative data about adult persons with a transfemoral amputation or knee-disarticulation with experience in using a prosthesis and focused on the users’ opinions. All eligible studies were independently screened by two reviewers. The results sections of the included studies were entered in Atlas.ti software (v8) and coded using the framework approach. The quality of the included studies was assessed using the Critical Appraisal Skills Program (CASP) qualitative research checklist. Results of the meta-synthesis were validated with prosthesis users (n = 8) in a focus group.

**Results:**

Out of 5757 articles, 14 studies were included. An overview of seven themes (‘prosthesis related’; ‘rehabilitation, costs and prosthetist’; ‘mental’; ‘physical’; ‘social’; ‘activities and participation’ and ‘walking’) containing 84 factors was created. Ten factors were added during the focus group, resulting in an overview of 94 factors that may influence the prosthesis use of lower-limb prosthesis users. Participants would like more user-involvement from the rehabilitation team. The development of a patient decision aid could help this process in the future.

**Conclusion:**

The large number of factors demonstrates that there is a great variety between prosthesis users and the factors that influence their prosthesis use. Therefore, it is important to take individual preferences into account for the selection of a new prosthesis.

## Introduction

In the Netherlands, there are roughly 10,000 persons who are using a lower limb prosthesis [1] in a total population of 15.000 persons with a lower limb amputation [2]. It is estimated that one third of this group has a transfemoral amputation (TFA) or knee-disarticulation (KD) [2]. Persons with a TFA or KD are heavily reliant on an adequate set of components for their prosthesis. A lower limb prosthesis consists of at least a prosthetic foot, a pylon, a prosthetic knee and a socket [2]. There is a large variety of prosthetic knees and feet available on the market and the technical developments of new prosthetic components are advancing at a quick pace. An important innovation in the field of lower limb prosthetics is the development of microprocessor controlled knee units (MPKs). An MPK contains sensors and a microprocessor that allows the knee to adjust automatically to the users’ intents during the stance and swing phase. The sensors capture data in real time and recognise which phase of the gait cycle the user is in. Based on this input, the movement of the knee can be adjusted (e.g. the swing phase can be slowed down, or the knee can be locked in position). Manufacturers suggest that this type of knee can offer more stability and safety for the user [3,4]. However, an MPK is two to ten times more expensive than conventional mechanical, non-microprocessor controlled knee units (NMPKs) [5,6]. Furthermore, the outcomes of various studies about the perceived benefits of MPKs over NMPKs are inconsistent [7–12].

The Dutch healthcare system revolves around integral care [13]. The goal of the integral care process surrounding lower limb prostheses is to achieve appropriate use. Appropriate use means that the prosthetic components are adjusted to the real needs and individual possibilities of the prosthetic leg user and are used efficiently. The rehabilitation team, consisting of a rehabilitation physician, physical therapist and certified prosthetist, determines the choice of the components for the lower limb prosthesis in consultation with the user. In the current care process, ‘stepped care’ is applied as much as possible: the cheapest device that seems to meet the requirements to reach the intended functioning, should be prescribed. If this device does not meet the needs of the user, a different and often more expensive variant is selected [13].

Based on our experience in daily practice, people with lower limb amputations seem to have increasingly higher expectations of their prosthesis, which are potentially due to the greater digital availability of information about the possibilities of newer prostheses. This information often tends to focus more on the advantages than on the disadvantages of innovative devices, such as MPKs. For example, there are many online videos of prosthesis users performing complex activities, such as mountaineering or skiing, with advanced prostheses. Videos do not often show users struggling with the usage of their devices. The introduction of technological innovations also tends to have the effect of an increasingly ‘shorter life span’ of the lower limb prostheses: users want to replace their old prosthesis before the replacement term has expired with a new, innovative one, because it seems more functional.

The choice for specific prosthetic components, such as the type of knee, may influence the users’ mobility [10,11,14,15], quality of life [7,9] and satisfaction [8,10,16] with their prosthesis and such a choice is an important decision. Due to a lack of scientific evidence, the choice for a certain type of prosthetic knee is often experience based instead of evidence based. The amount of previous qualitative studies that focused on prosthesis use from a user perspective is limited. Furthermore, most of these studies tend to focus on transtibial amputations or only a very small portion of the sample size consisted of KD or TFA.

To improve the process of adjusting the specific prosthetic properties to the expectations of the prosthesis users, it is of importance to first identify which factors have an influence on prosthesis use and could impact the users’ quality of life of the users and influence their satisfaction with the prosthesis.

Therefor the aim of this study was to identify factors that influence prosthesis use in adults with a TFA or KD.

## Methods

This study was carried out in two parts. First, a qualitative meta-synthesis was conducted to identify which factors can influence prosthesis use of adult lower limb prosthetic knee users. Qualitative results could originate from open questions in questionnaires, from interviews or from focus groups. The results of the meta-synthesis formed a pre-final framework of factors that might be of importance to prosthesis users regarding their prosthesis use. This pre-final framework was discussed during a focus group with users to assess whether the framework was correct and complete.

### Part I: Meta-synthesis

The meta-synthesis was registered in Open Science Framework [17]. To report the findings of the included studies in the meta-synthesis, the ‘enhancing transparency in reporting the synthesis of qualitative research’ (ENTREQ) guideline was utilised [18].

#### Search strategy and study selection

**Fig 1:**
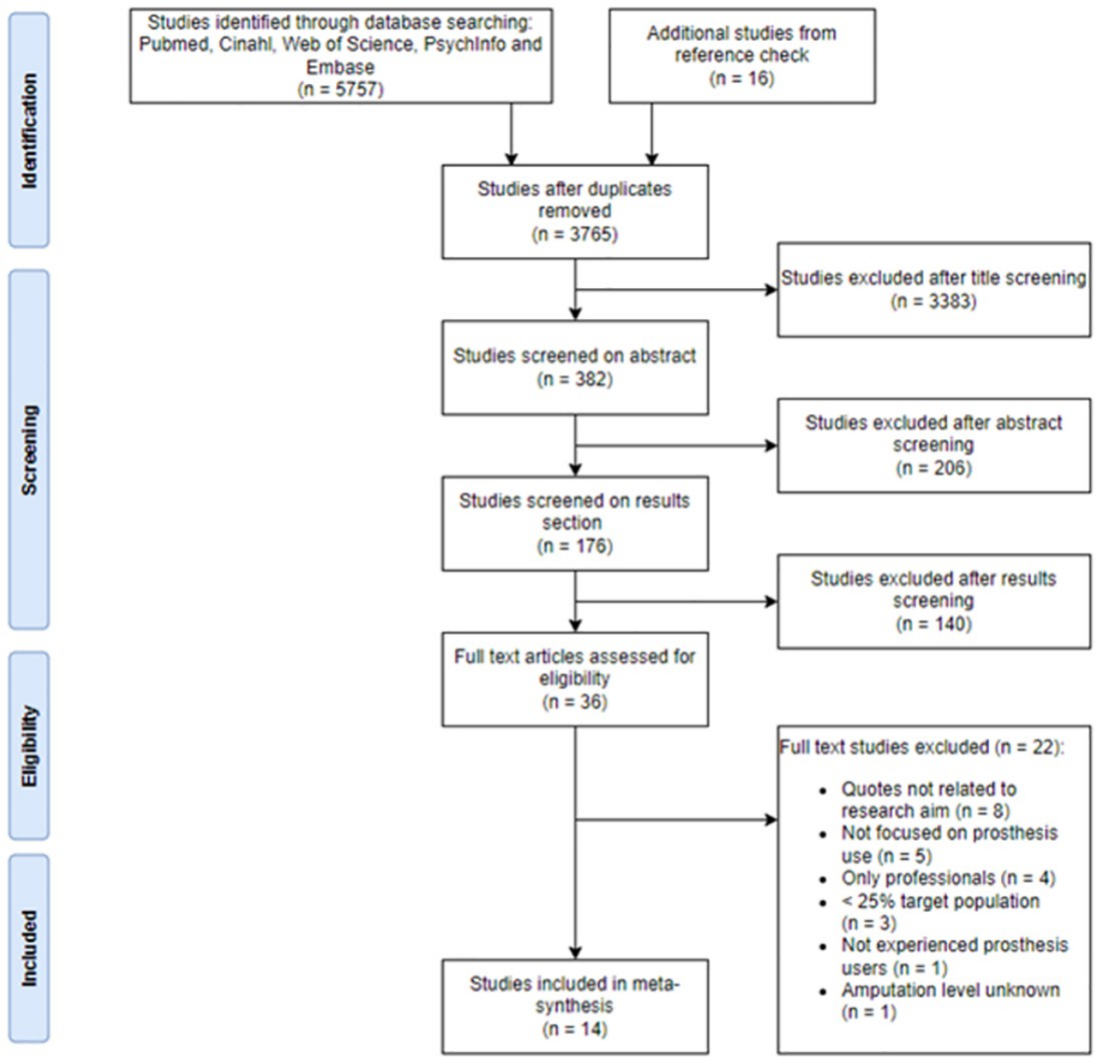
Preferred Reporting Items for Systematic Reviews and Meta-Analysis (PRISMA) flowchart for study selection.

In order to identify eligible studies, a search was conducted utilizing five electronic databases: PubMed, Web of Science, Embase, PSYCInfo and Cinahl. For this search, a search strategy was constructed together with an experienced librarian (S1 Text). We considered literature as eligible if it was not older than 25 years. The first commercially available MPK was released in 1997, therefor we only considered studies published after this date. Furthermore, we only considered studies that were published in Dutch or English. As can be derived from Fig 1, we initially searched for qualitative literature that contained data from experienced prosthesis users as well as healthcare professionals. However, during the screening process we decided to dedicate the study completely to the user’s perspective. Since the suggested influences from users and health professionals often did not align, we felt it would be more valuable to separate the two groups for analysis. For example, one of the included studies highlighted that independence was an important outcome for both users and health professionals. However, there was a disparity between what independence meant for the respected groups. For prosthesis it was a psychological outcome, but for health professionals it was a functional outcome [19]. The eligibility criteria can be found in Table 1. The initial search was conducted on March 4^th^ 2020 and an update was performed on January 20^th^ 2022.

**Table 1:** Eligibility criteria for meta-synthesis.

Inclusion	Exclusion
Participants ≥ 18 years old Knee-disarticulation or transfemoral amputation Experienced prosthesis users (> 1 year) Qualitative data/outcomes about prosthesis use, quality of life or satisfaction with prosthesis Published in Dutch or English	>25% of participants does not fit the inclusion criteria Osseo-integration Focus on 3D-printed or sports prostheses Published before 1997 No full text available Quotes not related to research aim

All eligible studies that were retrieved were merged into a reference management software system, EndNote (v20) [20]. Duplicates were deleted. The remaining studies were screened on title by the first author (CB). When there was any doubt about including the study based on the title screening, the study was included for the next round of screening. Following the title screening, two reviewers (CB & AV) independently screened the remaining studies for eligibility based on the abstracts. Subsequently, the results sections of the considered studies were screened independently (CB & AV) to ensure the gathered data was qualitative since this was not always clearly disclosed in the abstract. After the results sections were screened for eligibility, the full text of the remaining studies was read by the two reviewers. The reference sections of the included studies were checked for additional studies that could be considered. For all screening rounds, the agreement between the reviewers was calculated using Cohen’s kappa. In case of disagreement, the reviewers discussed until consensus was reached. Some of the included studies contained qualitative and quantitative outcomes or also included participants that did not meet the eligibility criteria. In these cases, only the relevant sections of the studies were used. Quotes from participants who did not meet the inclusion criteria were disregarded in the analysis.

#### Quality assessment

The quality of the included studies was assessed using the Critical Appraisal Skills Program (CASP) qualitative research checklist [21]. The checklist comprises ten questions, divided into three sections: ’are the results of the study valid’; ‘what are the results’ and ‘will the results help locally’. Two reviewers (CB & AV) independently conducted the quality assessment. Any disagreement was resolved through discussion in order to reach consensus. If no consensus was reached, a third reviewer (CS) was consulted and a final decision was made. Since the overall value of a qualitative study cannot be represented solely by the used methodology and how the results were reported, no studies were excluded based on the quality assessment [22].

#### Data extraction and analysis

The first reviewer (CB) extracted the following descriptive information from the included studies: participant demographics, study design, methods used for data collection and analysis and information needed to assess the quality of the studies. Subsequently, the results sections of the included studies were entered in Atlas.ti software (v8). The Atlas.ti software is used for qualitative data analysis [23] and offers the possibility to make use of different theoretical approaches, such as content analysis [24]. The text was coded with a directive content analysis approach [25], using the framework constructed by Kerver et al. [26]. This framework consisted of factors related to the use of upper limb prostheses. These factors were grouped in six different themes: ‘prosthesis related factors’, ‘rehabilitation, costs and prosthetist services’, ‘social’, ‘activities and participation’, ‘physical’, and ‘mental’. For the current study not all factors of the framework were considered during coding, since certain factors were specific to the use of upper limb prostheses. The factors related to upper limb prosthesis (e.g. grip strength, wrist control, etc.) were excluded. Initially we attempted to use open coding and using the categories or frameworks from the included studies. However, we felt these frameworks were not broad enough for the scope of this study, as they generally focussed on one aspect of prosthesis use. Subsequently, we searched for an overarching framework related to lower limb prosthesis use, but were unable to find a fitting framework. Since the framework by Kerver et al. [26] had a wide variety of categories and factors, this was the best-fitting framework, even though it focusses on upper limb prostheses. Two reviewers (CB & EV) independently coded the included studies and created new codes if quotes did not fit this preliminary framework. Afterwards the new factors were discussed until consensus was met. Newly identified factors on which consensus was reached were added to the framework and unused factors were removed in order to design the pre-final framework. Subsequently, all data were re-coded from blank to form the pre-final framework. Lastly, all factors were translated to Dutch in order to discuss these during a focus group with Dutch lower limb prosthesis users.

### Part II: Focus group

A focus group with persons with a TFA or KD was organised to check whether the pre-final framework that was created in the first part of this study was relatable and complete. Newly gained insights from the focus group were merged with the existing pre-final framework in order to form the final framework, which represents a complete overview of user-relevant factors that can influence the prosthesis use of persons with a TFA or KD.

The Medical Ethics Review Board of the University Medical Center Groningen (UMCG) judged that formal approval for the study was not needed (METc 2019/419). Prior to the focus group, all participants provided written informed consent.

The ‘consolidated criteria for reporting qualitative research’ checklist (COREQ) was used to accurately describe the results of the focus group [27] (S2 Table).

#### Participants

Due to COVID19-restrictions we were not allowed to invite participants from all regions of the Netherlands. Therefore, all invited potential participants were from the northern region.

Participants were considered eligible if they were adults with a unilateral TFA or KD who had at least one year of experience using a prosthesis. Based on these criteria, a purposive sample of eligible participants was selected by an experienced prosthetist. The eligible participants received an information letter and an invitation to take part in the focus group via mail.

#### Data collection

The focus group was organised in September 2020 and was held in a meeting room at the UMCG. All local COVID-19 regulations were followed during the preparation and execution of the focus group. The focus group was moderated by an expert in moderating focus group meetings (SvT) and took 90 minutes in total. The moderator was assisted by a female PhD student in lower limb prosthetics (CB) and a female medical student (EvV). The moderator and assistants were not acquainted with any of the participants prior to the focus group.

Before the start of the focus group, the participants were asked to sign the informed consent forms and fill out a short questionnaire about socio-demographic data (age, gender, side of limb loss, level of limb loss, time since limb loss, years of experience and type of knee). Subsequently, the moderator and assistants were introduced to the participants by stating their names, occupation and their role in the study. Shortly thereafter, the participants received a short introduction about the study and the purpose of the focus group. To start the focus group, the participants were asked two open questions: 1. *‘Which factors are of importance to you*, *as a lower limb prosthesis user*, *when choosing a prosthesis*?*’*; 2. *‘Which factors are important to you when using a prosthesis*?*’*.

Subsequently, the pre-final framework was presented to the participants per theme. All factors were written down on big sheets (Magic Charts), so the participants were able to see the overview. The moderator vocalised all factors and asked whether they were all clear and understandable. If necessary, additional explanations were given. The participants were then asked if they felt like some factors should not be part of the framework, or if they were missing factors. Suggestions for missing factors were written down on the sheets. Furthermore, they were asked if the presented themes were complete.

The focus group was audio recorded and transcribed verbatim. After transcribing the focus group, we wrote a summary about the focus group. This summary was sent to all participants, as well as the overview of the themes and factors. The participants were invited to respond with comments or corrections.

#### Data analysis

The transcript of the focus group was uploaded in Atlas.ti (v8) software and was coded with a directed content analysis approach [25], using the pre-final framework that was established in the meta-synthesis as a coding framework. Two female researchers independently coded the transcript (CB/EvV). Where necessary, new factors were added to the pre-existing themes. Any disagreements were resolved through discussion until consensus was reached. After the final framework was established, all data was re-coded from blank (CB). The final overview of the themes and factors, as well as illustrative quotes were translated into English (CB).

## Results

### Part I: Meta-synthesis

#### Study selection

A total of 5757 studies were identified during the initial database search. After deleting the duplicates and going through three rounds of screening, 36 studies were read in full text. Ultimately, 14 studies were included in the meta-synthesis (Fig 1). Cohen’s Kappa for the full text assessment was 0.83, which can be interpreted as a strong level of agreement [28].

#### Study characteristics

In total 375 participants from seven countries were included in the meta-synthesis (Table 2). Study sample sizes varied from seven to 92 participants. Five studies described participants who did not meet our inclusion criteria (transtibial amputation or lower), but could not be analysed separately [29–33]. Seven studies included participants with a bi-lateral amputation [19,29,30,32,34–36] and one study included a participant with a quadrilateral amputation [37]. Furthermore, two studies also included persons with upper limb loss [30,35].

**Table 2:** Summary of sample characteristics, used methodologies and quality assessment from included studies.

Study	Sample size	Age (years)	Gender	Origin of limb loss	Level of limb loss*	Time since amputation (years)	Country (ISO-code)	Data collection technique	Data analysis	CASP criteria unmet ^A^
Bragaru et al. (2013) [34]	26	Range: 21–77; Median: 60	7 F; 19 M	Vascular: 15; Trauma: 7; Oncology: 4	1 AD; 9 TT; 6 KD; 5 TF; 2 HD; 1 BL (TT/TF); 1 BL (TT/KD)	Median: 8,5; Range: 2–35	NLD	Interviews	Thematic data analysis	None
Bunce & Breakey (2007) [41] ^B^	10	Not reported	10 M	Not reported	Not reported	> 1 year ^C^	USA	Non-directive interviews	Not reported	4–9
Datta & Howitt (1998) [40]	22	Range: 25–76 Mean: 39.9	8 F; 14 M	Trauma: 16; Oncology: 5; Infection: 1	22 TF	Range: 5–53 Mean: 19.2	GBR	Semi-structured questionnaires	Not reported	6
Dunne et al. (2014) [32]	30	Range: 38–86 Mean: 63.80 SD: 11.63	4 F; 26 M	Vascular: 17; Trauma: 5; Other: 3	12 TT; 16 TF; 2 BL	Not reported	IRL	Semi-structured interviews	Inductive thematic analysis	None
Gallagher & MacLachlan (2001) [29] ^B^	14	Range: 20–50	8 F; 6 M	Not reported	5TF; 7TT; 2BL	Not reported	IRL	3 focus groups with 4/5 participants	Inductive thematic analysis	4, 6, 7
Hafner et al. (2016) [36]	37	Range: 22–71; Mean: 50.4; SD: 12.5	11 F; 26 M	Trauma: 25; Infection: 11; Vascular: 3; Other: 3 Oncology: 2;	20 TT; 8 TF; 1 HD; 5 BL (TT/TT); 1 BL (TK/TF); 1 BL (TT/KD); 1 BL (TF/HD)	Range: 0.6–60.5 Mean: 14.7 SD: 14.8	USA	4 focus groups	Inductive thematic analysis	7
Järnhammer et al. (2018) [38]	16	Range: 21–67; Mean: 38	6 F; 10 M	Trauma: 11; Oncology: 2; Infection: 2; Other: 1	11 TT; 1 KD; 4 TF	Mean: 10 SD: 9	NPL	Semi-structured interviews	Qualitative content analysis	None
Jefferies et al. (2018) [35]	24	Only reported for interviews: Range: 18–62; Mean: 43.89; SD: 12.66	5 F; 19 M	Acquired: 17; Congenital: 5	13 LL (2BL); 10 UL; 1 BL (LL/ UL)	Not reported	IRL & USA	Unstructured interviews; published autobiographies, weblogs and internet discussion forums	Iterative and incremental	6, 7
Legro et al. (1999) [33] ^B^^F^	92	Range: 22–81; Mean: 55	13 F; 79 M	Not reported	8 AD; 57 TT; 3 KD; 23 TF	> 1: 35; >5: 57	USA	Questionnaire and open-ended questions	Thematic analysis	8
Norlyk et al. (2016) [39]	8	Range: 33–74; Median: 53.5	2 F; 6 M	Vascular: 4; Trauma: 2; Infection: 2;	4 TT; 4 TF	Not reported	DNK	Interviews	Inductive thematic analysis	None
Schaffalitzky et al. (2011) [19] ^H^	24	Range 29–86; Mean: 63	11 F; 13 M	Vascular: 9; Trauma: 8; Oncology: 3; Infection: 3; Congenital: 1;	13 TT; 8 TF; 1 BL (TT/TF); 2 BL (TT/TT)	≤10: 13; 11–20: 1; 21–30: 3; 31–40: 2; 41–50: 3; 51–60: 1; 61–70: 1	IRL	Focus groups	Inductive thematic analysis	6
Senra et al. (2012) [31] ^D^	42	Range: 22–82; Mean: 61; SD: 13.5 22–45: 4; 46–64: 18; ≥65: 20	7 F; 35 M	Vascular: 35; Oncology: 4; Trauma: 3;	4 AD; 22 TT; 16 TF	Range: 0.3–17; Mean: 2.3 SD: 3.3 ≤ 1: 25; 1–5: 14; >5: 3	PRT	Semi-structured interviews	Thematic and categorical analysis (by Bardin)	None
Tran et al. (2020) [37] ^G^	7	Not reported	5 F; 2 M	Trauma: 4; Vascular: 1; Infection: 1; Congenital: 1	4 TT; 2 TF; 1 QL (TT/PF/TR/PH)	<1: 1 ≥10: 6	USA	Focus group	Thematic analysis	None
Waldera et al. (2013) [30] ^C^	23	Only reported age at amputation <20: 2; 20–29: 4; 30–39: 1; 40–49: 3; 50–59: 1; ≥60: 1; Unknown: 11	Not reported	Trauma: 16; ‘Other’: 4; Vascular: 2; Unknown: 1	15 TT; 1 KD; 6 TF; 1 BL (TT/TF)	≤10: 5; 11–20: 5; 21–30: 2; 31–40: 9; 41–50: 2	USA	Interviews (phone or in person)	Inductive thematic analysis	5, 7

^A^ CASP criteria: (1) Was there a clear statement of the aims of the research?; (2) Is a qualitative methodology appropriate?; (3) Was the research design appropriate to address the aims of the research?; (4) Was the recruitment strategy appropriate to the aims of the research?; (5) Was the data collected in a way that addressed the research issue?; (6) Has the relationship between researcher and participants been adequately considered?; (7) Have ethical issues been taken into consideration?; (8) Was the data analysis sufficiently rigorous?; (9) Is there a clear statement of findings?; (10) How valuable is the research?.

^B^ Data-collection consisted of questionnaires (n = 42) and interviews with a different participant group (n = 10). Only the results of the interviews are included.

^C^ Participants had at least one year of experience using a C-leg and had used a NMPK prior to C-leg.

^D^ Sample consisted of amputees with several levels of limb loss, but their quotes could not be distinguished for analysis.

^E^ Data was collected with the Prosthesis Evaluation Questionnaire (PEQ) and three open questions at the end. Only the data from the open questions are included.

^F^ Study consisted of six focus groups with prosthesis users and ten interviews with service providers. Only the focus group results are taken into account.

^G^ Not all participants used prostheses.

^H^ Study consisted of two focus groups; one with prosthetists and one with prosthesis users. Only the latter is included in this table.

^I^ Study included both persons with upper limb loss and lower limb loss. Only parts about lower limb loss have been included.

* Levels of limb loss are presented from distal to proximal.

ISO-code, country code assigned by the International Organization for Standards; CASP, The Critical Appraisal Skills Programme qualitative research checklist; SD, standard deviation; F, female; M, male; TT, transtibial; TF, transfemoral; AD, ankle-disarticulation; KD, knee-disarticulation; HD, hip disarticulation; BL, bi-lateral; QL, quadri-lateral; PF, partial foot; TR, transradial; PH partial hand; IRL, Ireland; USA, United States; NLD, Netherlands; DNK, Denmark; PRT, Portugal; GBR, United Kingdom of Great Britain; NPL: Nepal.

#### Quality assessment

The quality of all included studies was assessed using the ten questions from the CASP qualitative checklist. If a question was answered with ‘no’ it meant the criterion was unmet (Table 2). Six studies did not have any unmet CASP criteria [31,32,34,37–39], four studies had one unmet criterion [19,33,36,40], two studies had two unmet criteria [30,35] and two studies had three or more unmet criteria [29,41].

#### Findings of meta-synthesis

The meta-synthesis of the 14 included studies resulted in a framework of seven themes, containing a total of 84 factors. In addition to the six themes determined by Kerver et al. [26], a seventh theme, ‘walking’, was added to form the pre-final framework (S3 Table).

### Part II: Focus group

#### Participant characteristics

A total of 18 eligible participants were approached by letter, of whom ten agreed to participate. On the day of the focus group, two participants unfortunately cancelled. Furthermore, two participants ultimately did not meet all inclusion criteria, as one had a transtibial amputation and the other had an osseo-integration. They did participate in the focus group, but their quotes were not included in the final analysis. The group consisted of 6 male and 2 female participants. The mean age was 60.6 years old (range: 40–78 years; Table 3).

**Table 3:** Demographics of focus group participants.

Participant	Age	Gender	Amputation level	Years of experience using prosthesis	Type of knee
**1**	54	M	TF	39	MPK
2*	78	M	TT	3	-
**3**	53	F	TF	1	NMPK
**4**	77	M	TF	6	NMPK
**5**	40	F	TF	1	NMPK
**6**	61	M	TF	57	NMPK
7*	60	M	TF	7	MPK OI
**8**	62	M	TF	3	NMPK

M, male; F, female; TF, transfemoral; TT, transtibial; MPK, microprocessor controlled knee; NMPK, non-microprocessor controlled knee; OI Osseo-integration.

* Participant did participate in focus group discussion, but quotes were excluded for analysis.

#### Findings focus group

**Fig 2:**
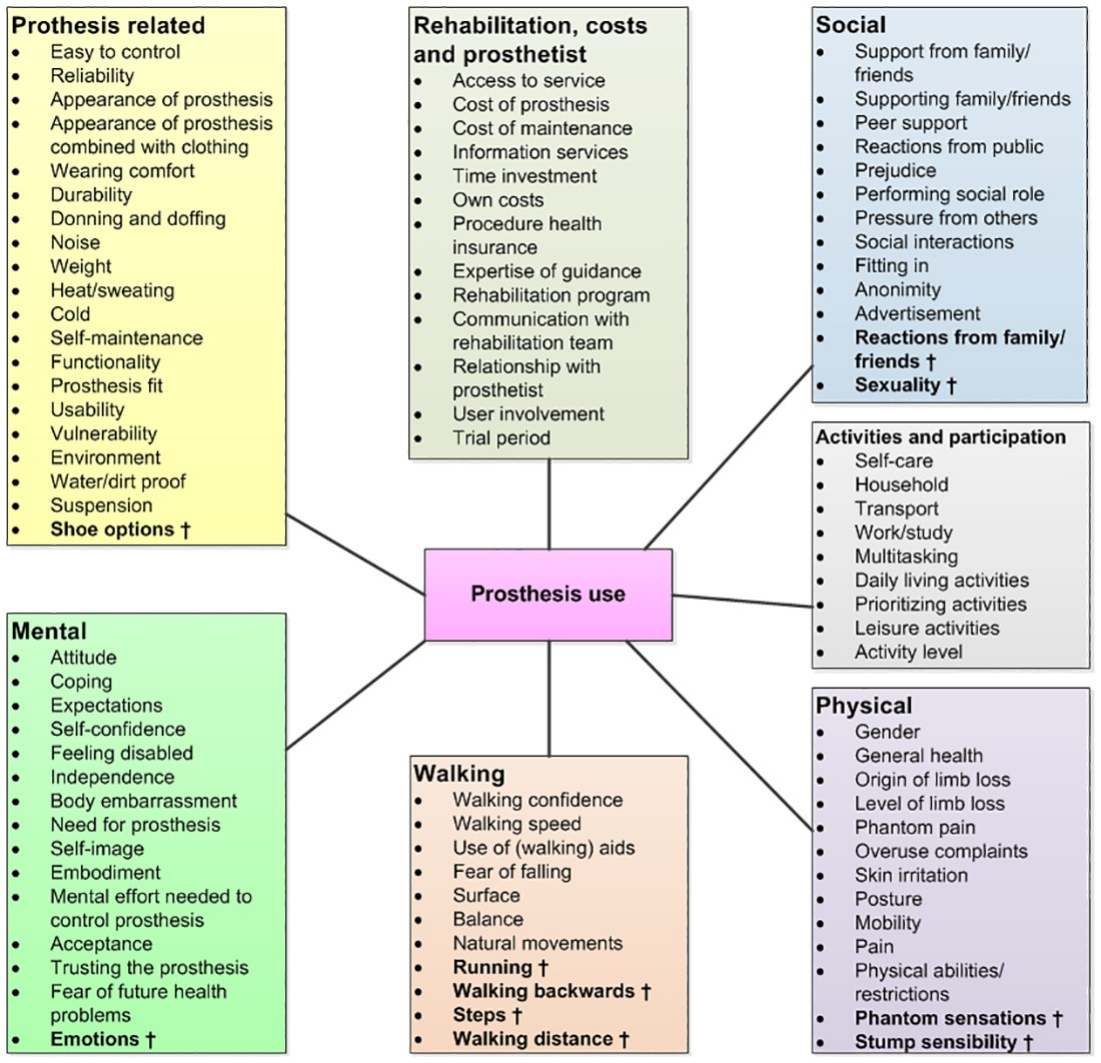
Overview of the themes and factors of the final framework †added during focus group.

The participants agreed with the seven themes of the pre-final framework but had 14 suggestions for additional factors. Since four had a clear overlap with existing factors, eventually ten factors were added to construct the final framework (Fig 2).

#### Themes and factors

**Theme 1: Prosthesis related**

This theme contains factors related to the characteristics of the prosthesis.

***Meta-synthesis***

The appearance of the prosthesis was often mentioned as an important factor [19,29,33,35,37,41]. While most of the participants preferred the prosthesis to blend in and not be noticed easily, some participants did not mind about the appearance of the prosthesis as long as it was functional.

*“After it happened to me and I had got the leg*, *this monstrosity of a thing with hinges and everything*… *You think oh my God look at that thing*. *But then you start to move and you realize that you are up and moving again*. *[*…*] I didn’t give a damn who was looking at me*, *I just wanted to get out and about”*

[29]

The appearance of the prosthesis in combination with clothing was also discussed a number of times, since clothing can often conceal the physical impairment [19,29].

Participants mentioned that the wearing comfort and the fit of the prosthesis, specifically the socket, can have a great impact on their prosthesis use and satisfaction with the prosthesis [19,29–31,35–37]. It was reported that an improperly fitted prosthesis was the most common cause of stump pain [29].

*“It is very frustrating*. *Sometimes you can get them and you can walk for miles and they will be grand*. *The next day you could put on the limb and it will start cutting you*… *It is the most annoying part*.*”*

[29]

Three articles mentioned the importance of reliability and the frustration that comes with malfunctioning prostheses [29,30,33]. A malfunctioning or broken prosthesis left the users immobile and dependent on others [29].

*“It happened to me at work one day when the knee went on it [knee buckled]*. *I just couldn’t move*. *Two fellas had to carry me out into another fella’s car to give me a lift home*. *The thing broke and you couldn’t move*.*”*

[29]

The durability of the prosthesis can have an effect on a person’s work, activities and number of visits to the prosthetist for repairs [30,33,40]. Therefore all participants who mentioned this factor expressed an interest in durable prostheses.

***Focus group***

While discussing this theme, participants mentioned that reliability is an important factor to them. The prosthesis can sometimes act unpredictable or break down suddenly, which results in potential hazards and immobility for the user. Participants also mentioned that the fit of the prosthesis influences their prosthetic use and satisfaction. Fluctuations in the residual limb were mentioned as a reason for changing the fit of the prosthesis. Changes in the weather and temperature were discussed as possible explanations for these fluctuations.

*“Everything depends on the socket*. *As soon as the socket doesn’t fit correctly*, *you are continually slowed down*.*”*

*[P3]*

The participants also discussed the weight of the prosthesis. It was mentioned that the weight is not only an issue while wearing the prosthesis, but also when it is taken off.

*“To me*, *the weight is not only decisive when I’m wearing it*, *but also when I’m not*. *With cleaning and things like that*, *I do think mine [prosthesis] is very heavy*.*”*

*[P3]*

One of the participants also mentioned that even though the weight of the prosthesis is roughly the same as a healthy leg, it does feel heavier.

Lastly, the ability to change between multiple types of shoes, as well as the properties of the shoes were discussed, which resulted in the addition of the factor ‘shoe options’ to the final framework. It was discussed how the weight, stiffness of the sole and height of the heel could influence the wearing comfort of the prosthesis. Furthermore, female participants expressed the desire to be able to change the heel height of their prosthesis in order to wear heels.

*“I find it annoying that you can’t buy the shoes you want*. *They can’t be too slippery and I always have to do something about the heels*.*”*

*[P1]*

**Theme 2: Rehabilitation, costs and prosthetist**

The theme consists of factors that are related to each aspect of rehabilitation after an amputation, receiving a prosthesis and the additional costs.

***Meta-synthesis***

Some participants experienced the rehabilitation program as an inadequate or insufficient resource [31]. However, the majority of the participants expressed their satisfaction about their rehabilitation program. It was a way to learn new skills and train with their prosthesis [30–34,36–39].

*“Rehabilitation is important because it is helping me to return to my daily life and most importantly to walk again*! *I have hope in the prosthesis*! *It will help me become a different person and leave the wheelchair*.*”*

[31]

Participants also had contradictory opinions about the expertise of guidance they received. Whereas some were enthusiastic about the rehabilitation staff others expressed that they felt like their prosthetist did not listen or lacked proper training [30].

*“Rehabilitation has been very helpful for me*… *I am able to walk alone again*! *The staff has been lovely and I’m feeling like another person*!*”*

[31]

***Focus group***

Throughout the focus group meeting, one factor that kept coming back into conversation was the health insurance procedure. Participants expressed a lot of frustration about rejections for certain prosthetic components and in one case being denied a trial period to test a specific prosthetic knee for a few weeks.

*“Something that was missing for me was the ability to test [different components/prostheses]*. *I think it is essential*. *You can want all kinds of things*, *but if you notice it isn’t possible or it does not suit you*…*”*

*[P3]*

Another participant mentioned that he was given one type of knee and had no other options.

*“It was clear that I could just get this knee*. *It did not come up that anything else was possible*. *They [health insurer] thought I managed well enough on this mechanical knee*.*”*

*[P8]*

Overall, the participants expressed the desire to have a clear, transparent communication between them and the rehabilitation team. The opinions about the user involvement in the process of choosing a prosthesis were mixed. Other participants did not recognise such experiences and explained that they were involved in the process by the rehabilitation team. They also felt supported in their ‘battle’ with the health insurance company. Further along in the conversation, the importance of having a good relationship with the prosthetist was emphasized.

*“Well*, *the most important thing is to have a prosthetist who understands what you need and how to go down those paths*. *But in addition to that*, *you have to have a health insurance company that grants it to you*, *because it’s just a grant-factor*…*”*

*[P5]*

Something that the participants felt was missing, was available information about different types of prostheses and prosthetic components. They have to rely on the information they receive from the rehabilitation team, since the information online is very limited and subjective.

*“I mentioned it from the beginning*, *you [rehabilitation team] can tell me what I need*, *but how am I supposed to know*? *I have to believe what you tell me*, *because there is no information available*. *The problem is that there’s not enough information to find about this [prostheses]*. *… Yes you can find a lot about the C-leg or Rheo knee*, *but that’s it*.*”*

*[P5]*

**Theme 3: Social**

This theme consists of factors related to a person’s social relationships, their position in society and the associated reactions.

***Meta-synthesis***

Having a good support system in place can help prosthesis users with several aspects of life; for example: processing the amputation, dealing with finances and helping with tasks in and around the house. Receiving support from friends and family was often discussed, however not all participants specified what support they received. Friends and family mainly supported the prosthesis user with the acceptance of their situation [29–34,36,38].

*“My family is the main reason for my recovery*! *They have been giving me a lot of support*, *helped me to walk again and to cope with this situation*.*”*

[31]

Being able to talk to other persons with a lower limb prosthesis also had a positive influence on the participants. The presence of peer support was a good experience for the participants. Those who did not experience it during their rehabilitation phase expressed missing this, since the rehabilitation team can help but does not understand the full situation [29,32–34,38].

*“I feel support groups are extremely important*. *Doctors and therapists are also important*, *but they cannot understand the frustrations an amputee goes through unless they are an amputee*.*”*

[33]

Most participants mentioned a fear of getting negative reactions in public, or having experienced this in the past [19,29,31,32,34,35,39,41]. One participant mentioned being torn between wanting people to know about the prosthesis and not wanting to tell them.

*“Yeah*, *but I am always more comfortable if people know*. *I have been in so many situations for example in pubs where people might slap my leg and say Jesus what is that*. *So I would be more comfortable if people knew*. *At the same time I don’t go broadcasting it*.*”*

[29]

Many participants expressed a desire to fit in with other groups and be the same as others [19,29,34,35,38,39,41]. They find it hard to accept that they stand out and want to belong and ‘be normal’, which often means wanting to be the same as persons without lower limb loss.

*“I can ambulate quite effectively on it and I don’t feel so cut-off or left out of what everybody else is doing*. *And that’s really a big thing for me*, *because I felt like I didn’t belong or that I was substandard somehow as everyone moved about freely and around me*. *And that was difficult to accept*, *it was hard on me*.*”*

[41]

***Focus group***

At first glance, the factor ‘advertisement’ was not clear to all participants. Even after a further explanation, one of the participants still did not relate having any influence on his prosthesis use or satisfaction to this factor. Others mentioned that the information on the internet was often focussed on the more expensive prostheses.

*“When you are looking for information*, *you only find the very expensive prostheses to which the health insurance companies say no*.*”*

*[P5]*

Subsequently, the participants discussed the different reactions they get from others in public. The experiences varied from positive to negative experiences.

*“I’m in shops more often now and often I have the mobility scooter with me*, *because I can’t walk a good distance yet*. *I notice that people find me very annoying*, *or I’m too slow*, *or you hear a sigh*…*I find it difficult*.*”*

*[P3]*

Anonymity was a topic that was shortly discussed and the opinions varied. Whereas a few participants did not mind showing their prosthesis in public, others did not feel comfortable doing so and preferred not to be noticed.

*“Other people just walk around without any clothes on [covering the prosthesis] and have no problem with that; I don’t have that (or I don’t feel that way)*. *The sound [of the prosthesis] was also annoying to me*. *Basically I just want to be unobtrusive*.*”*

*[P1]*

Most participants also mentioned a variety of reactions they had received from friends and family, ranging from prejudice about their abilities to problems with acceptance. This could sometimes lead to unwanted help and frustrations. One participant mentioned that it sometimes felt like his family had a harder time accepting his amputation than he did. Since the participants felt that these experiences did not fit in any of the factors on the pre-final framework, the factor ‘reactions from friends/family’ was added.

Wearing a prosthesis can have an effect on someone’s ability to perform specific roles, such as being a parent.

*“When my daughter was born*, *I was like*: *you can’t walk around with this [prosthesis] anymore*. *You can’t hold a baby and risk collapsing*. *So then I got a C-leg and I am very happy with it*.*”*

*[P1]*

It can also influence the way they interact with their loved ones.

*“I feel like a different person when I’m wearing a prosthesis*. *It may sound a bit crazy*, *but I feel more complete when I am standing up*. *Life is so much more fun standing up*. *Being able to hug your husband or looking someone in the eyes*.*”*

*[P3]*

Lastly, one participant noticed the absence of the factor ‘sexuality’. Other participants agreed that this was an important factor, but it was not discussed any further. Therefore, ‘sexuality’ was added to the final framework.

**Theme 4: Activities and participation**

Factors included in this theme are related to a person’s personal tasks and activities, as well as their participation in society.

***Meta-synthesis***

Being able to participate in leisure activities, such as sports, are often mentioned as a positive influence on participants’ life [29,31,32,34–36,38–40]. Furthermore, the use of a prosthesis can help with other aspects as well, like returning to school or work and self-care [29–32,34–36,38–40].

*“Today I’m a new person*! *I returned to school and to work*! *I bodyboard*! *My life is not the same but it [amputation and prosthesis] is already a part of me*! *I adjusted my life to it*, *but I haven’t stopped doing my favourite hobbies or meeting my friends*.*”*

[31]

Participants also discussed the fact that the use of a prosthesis can sometimes force them to prioritize certain activities over others, in order to preserve their energy or because of discomfort [32,34–36].

*“First of all*, *you [do] less activities than you would normally because*, *say like you planned to go to the mall or you planned to go to the beach or something and you are having a bad day or in pain with your stump*. *Of course you wouldn’t do it that day and you would be a little grouchy*, *you know*, *because the pain is irritating and you would be less active in your normal day*.*”*

[36]

***Focus group***

One of the participants mentioned that he felt that the rehabilitation team and prosthetist should pay more attention to leisure activities. Other participants agreed with this statement and spoke up about the desire to partake in sports. Some of them felt held back by the rehabilitation team, since they were told they could no longer do certain sports or would have to rely on a wheelchair to do so.

*“What I miss very much about this is that*, *in practice*, *hobbies are not considered at all*. *In fact*, *I want to do a lot of sport*, *but in my case they said*, *yes*, *do that in a wheelchair*. *So I do indeed miss that*, *if you want to*, *you just don’t get the opportunity*. *That hobbies are looked at*…*I think as a luxury”*

*[P3]*

Prioritizing activities was something all participants agreed on. Due to the use of the prosthesis, they had to make decisions about which activities they could or could not do. The main concern was running out of energy faster.

*“If I choose to go for a walk*, *just to train myself*, *then that is a choice*. *I will put my energy into that and afterwards I need some time to recover*.*”*

*[P1]*

Overall, most of the participants agreed that they shared the desire to be able to do things the way they used to before their amputation.

*“I want to be able to do the things I would normally do [before amputation] with my prosthesis*. *I want to be able to walk a bit faster with my children and just do my daily things in a normal way*.*”*

*[P5]*

**Theme 5: Physical**

This theme includes all factors that are related to the body of the prosthesis user.

***Meta-synthesis***

Participants had mixed opinions about the physical abilities and restrictions that are connected to prosthesis use. While some where happy to be able to walk and have some mobility, others highlighted some physical restrictions they experienced as limitations such as not being able to walk up the stairs or to run.

*“My biggest problem was teaching my daughters about riding a bike and running alongside them for a long distance … I could run a few feet*, *but wasn’t running a block or anything like that*.*”*

[36]

The occurrence of skin irritation was a problem for many prosthesis users [29,33–37]. One participant acknowledged some limitations, but opted not to focus on these:

*“Yes*, *there are limitations*, *even though I choose not to focus on them*. *I don’t have the same endurance I once did*. *I can’t run as fast as I would with a real leg*. *Sometimes*, *I still get blisters*, *or lose my balance*. *But I look at it from the perspective that everybody has bad days*. *Everyone sits down and cries once in a while*. *And that’s okay*.*”*

[35]

***Focus group***

One of the first things that was mentioned when this theme was discussed was the factor ‘gender’. At first it was not clear to all participants what this factor entailed, but two participants described gender-specific problems they experienced while using their prosthesis. For women this entailed having to cope with menstruation. For men the experience of getting one’s testicles stuck in the brim of their socket was discussed as a negative gender-specific problem.

The next topic that was discussed, was the sensibility of the stump. Scenario’s that normally would not hurt, suddenly felt excruciating. Since this topic did not fit any of the existing factors, ‘stump sensibility’ was added.

*“The other day when I was running the fan in the summer*, *it felt like my whole leg was on fire*.”

*[P5]*

In addition to stump sensibility, skin irritation was discussed as an issue that can have an impact on the prosthesis use.

*“I’ve been walking with a prosthesis for 40 years and since a few years I have had problems with my skin*. *It was never an issue*, *but now it is and it bothers me*. *I can no longer walk as much as I want*.*”*

*[P1]*

Subsequently, the participants explained that the factor ‘phantom sensations’ was missing from the overview, since this was very different from phantom pain. Therefore, ‘phantom sensations’ was added to the final framework.

**Theme 6: Mental**

The factors in this theme are all related to the prosthesis user’s thoughts and feelings.

***Meta-synthesis***

Regaining independence after amputation and not having to rely on the help of others was an often recurring factor of interest [19,29–34,38,39]. One study mentioned that independence should not be observed solely as a functional outcome, since it also is related to psychological aspects.

One participant explained the effect of being dependent on others:

*“I can’t accept this situation because it’s revolting being like this*… *without driving*, *walking*, *working*… *it’s very sad depending on others*. *“*

[31]

Undergoing an amputation and becoming dependent on others can also have an impact on someone’s self-image.

*Today I can’t do many things*, *I became more dependent on others*, *with less autonomy and I changed myself*! *Today I’m a more nervous and explosive person*… *I liked reading and today I don’t*… *many things changed for me*.

[31]

The use of a prosthesis can have a positive effect on a person’s independence.

*“Until I started using the prosthesis*, *I was more dependent on others*… *However I still need a lot of help*.*”*

[31]

The last factor that was mentioned several times, was the importance of having a positive attitude [19,29–33,35,36].

*“First*, *you must have a good attitude*, *otherwise*, *you won’t use the device*. *We should do the best with what we’ve got and have faith*.*”*

[30]

For some it was hard to have a positive attitude, since they had a hard time coping with- and accepting their amputation and prosthesis use [29–35,37–39]. On the other hand, there were also participants who had accepted that even though they might no longer be able to do everything, they were at least going to try.

*“If I was given a challenge I would try anything*, *I would never say no I can’t do that or I won’t do that*. *I would try it to the best of my ability*, *if I can do it I will do it*, *if I can’t*, *I can’t*. *I will try it and maybe I can’t go this far*, *it might be only to go that far but at least I will try it*.*”*

[32]

***Focus group***

The factor ‘need for prosthesis’ was discussed first and most participants agreed that while they technically could do without their prosthesis, they felt less comfortable if they did not wear it.

*“Still*, *I think it’s an interesting point*, *because if I don’t put it on one day because it bothers me too much [stump sensibility]*, *then I don’t like it*. *Then I think that*, *um*, *not only does it make it harder to move around the house*, *but it just bothers me*. *So yes*, *in that sense*, *I can’t do without them*.*”*

*[P1]*

Finally, a participant mentioned that a number of emotions was already represented in the overview, but that he missed the factor ‘emotions’. Other participants agreed that this theme should include emotions.

*“Sometimes I get extremely annoyed about my leg and that actually just makes it worse… (Angry emotions)*.*”*

*[P8]*

**Theme 7: Walking**

This theme consists of factors related to walking with a lower limb prosthesis.

***Meta-synthesis***

Fear of falling was the most often mentioned factor and a lot of participants mentioned that this fear influenced their daily life activities [19,29,30,34,36,39]. They were aware of the fact that if they did fall, it would have a large impact on their health and mobility. Participants also mentioned that they always have to focus on the surface they walk on, as this is more difficult when using a prosthesis [30,36,40].

*“My first priority is always know where I’m stepping*. *[Amputees] tend to walk a little slower and damn straight*. *If we don’t*, *we find [we fall] more often than we like to claim*.*”*

[36]

***Focus group***

Due to the focus group, four factors were added to the theme ‘Walking’ in the final framework. One participant immediately noticed that walking distance was missing. Other participants agreed that it should be added. The next factor that was discussed was running and the need for a special sports prosthesis. One participant mentioned he ran on his regular prosthesis once.

A following factor was ‘steps’; the ability to go up or down small obstacles or stairs. The researcher asked whether this was the same as the walking surface, but participants said it was a different factor and it should be part of the final framework.

*“To me*, *it is essential to be able to go up and down steps while walking*.*”*

*[P3]*

Subsequently the participants discussed natural movements and walking confidence. Even though a few participants mentioned that a natural looking gait was important to them, one participant said walking confidence was more important to her.

*“When you are confident when you walk*, *it doesn’t matter if you can walk very neatly or with a limp*. *As long as you feel good (about it)*.*”*

*[P5]*

Fear of falling was something that was an issue for a few of the participants. One of them expressed he constantly had to focus not to fall while walking.

*“I would like for once to be able to walk without having to focus all the time; without a fear of falling*.*”*

*[P8]*

The final factor that was added to this theme, was the (in-)ability to walk backwards.

## Discussion

The meta-synthesis of qualitative literature yielded an interesting overview of user-relevant factors that have been researched so far. The final themes were congruent with the themes that were formed for upper limb prosthesis users [26], except walking. However, the results of the focus group illustrated that certain factors are under-represented in qualitative literature, even though they are of importance to prosthesis users. Ten factors were added to create a complete overview of all user-relevant factors regarding lower limb prosthesis use. These factors were: ‘shoe options’, ‘reactions from friends/family’, ‘sexuality’, ‘phantom sensations’, ‘stump sensibility’, ‘emotions’, ‘running’, ‘walking backwards’, ‘steps’ and ‘walking distance’,. These factors may have only been studied in quantitative studies and not in qualitative studies, which may explain this lack of information.

Many of the mentioned *prosthesis related* factors (e.g. comfort, weight, fitting and reliability) have been mentioned many times before in literature [16,42]. Although these problems seem to be straightforward, they have apparently not been solved yet and remain to be an issue for lower limb prosthesis users. Being able to self-adjust parts of the prosthesis is a valuable addition of the focus group: e.g. being able to adjust the prosthesis when you would like to wear others shoes. Adjustable prosthetic feet are commercially available, but they tend not to be used as often as non-adjustable prosthetic feet.

Most physical issues (e.g. gender specific issues or skin-irritation) had to do with discomfort of the prosthesis. There are several developments that can aid in these situations, but they come with their own issues. For example, discomfort issues regarding a hard socket might be solved by using a different type of socket or osseo-integration [43].

Based on the results of the focus group and literature review, it is safe to say that the rehabilitation treatment could be improved on a few fronts. The main issue participants seemed to have within this theme was the lack of user involvement in rehabilitation training and in the choice for different prosthetic components. Furthermore, they also felt they lacked information about this process. There should be more focus on a shared decision making process, in which the prosthesis user is more involved. Various studies [44,45] have shown that shared decision making can help patients become more knowledgeable about different treatments, help them to assess the risks and benefits of these treatment options, and reduce feelings of conflict and irritation about feeling misinformed.

Being independent from others, having a positive self-image, having a positive attitude and being able to cope were most mentioned. If a psychologist gets involved in the treatment of the individual with an amputation, these factors could be addressed during the rehabilitation process. Furthermore, the value of peer support was discussed in both the literature and the focus group. Previous studies have shown that contact with peers can help to put their own experiences into perspective, be a credible source for information [46], and can be beneficial for the prosthesis user to make informed decisions when it comes to their prosthesis [47]. A few rehabilitation centres in the Netherlands have already implemented some form of peer support in their rehabilitation training and it would be interesting to look into the effect of this in future research.

Since the fear of falling and lack of confidence was apparent, it is important to inform prosthesis users on the higher risk of falling and teach them how to decrease the chance of injury when they fall [48]. Therefore, rehabilitation training should also include fall training in different circumstances and different surfaces. Previous studies suggested that administering fall prevention training may reduce falls in prosthesis users [49,50]. Furthermore, it has been shown that a lack of confidence in balance may lead to the avoidance of activities and prosthesis use [51,52]. Therefore, fall prevention training should also include specific balance exercises.

### Healthcare in the Netherlands

All persons working or living in the Netherlands are obliged to buy a basic health insurance from a private health insurance company (HIC) and they can also opt to take out a supplementary insurance [53]. By law, all prosthetics and orthotics (P&O) services should be covered by the basic insurance as long as there is a valid prescription from a medical specialist. All HIC sign new contracts with P&O companies each year formalizing the budgets for every possible orthopaedic device. The basic insurance covers the prosthesis that the HIC deems most adequate for the user. When the rehabilitation team and user agree on a more advanced or different type of prosthesis (e.g. a sports prosthesis or MPK), it has to be approved by the HIC first. Since the Dutch healthcare system is different from most other countries, not all data found in international literature can be directly applied to the Dutch situation. This might explain why factors such as ‘cost of prosthesis’, ‘cost of maintenance’ and ‘own costs’ were often found in international literature, but barely discussed during the focus group. The factor ‘procedure health insurance’ on the other hand was discussed at length. Most participants expressed frustration about the approval procedure they had to go through when asking for a new type of prosthesis or prosthetic component. There was a lot of uncertainty about the application process and a few participants mentioned that it sometimes felt like it depended on the mood of the HIC employee whether their application was approved, rather than on clear guidelines. It became clear that this is a big obstacle and source of frustration for lower limb prosthesis users, mostly because it lacks transparency. Therefore, it would be helpful to look into this process in the future and create more transparent guidelines.

### Future research/clinical implications

The final overview of factors that was formed during this study gives us a complete overview of factors that may be of importance when using a prosthesis, which can be used in future research and can be implemented in prosthesis related healthcare. The broad overview illustrates a great variation of factors that could influence prosthesis use. Is can be difficult for prosthesis users to keep all of these factors in mind during a conversation with the rehabilitation team. The created overview can help as a conversation-guide. Furthermore, our research team will take the results of this study as input for the development of an online patient decision aid (PDA) that will assist prosthesis users and their rehabilitation teams when choosing a new prosthesis. This PDA will be written for persons with a TFA or KD and will inform them about the pros and cons of several prosthetic components. By using the PDA, the user might feel better informed and feel more included in the decision process [54]. Furthermore, the PDA can be a useful tool to use during conversations between the user and members of the rehabilitation team and improve the shared-decision making process [45].

### Strengths and weaknesses

The main strength of this study was the fact that the users’ perspectives were taken into account. The findings of the meta-synthesis were supplemented by opinions of lower limb prosthesis users.

A downside to the focus group was that it was a rather small sample size. We aimed for a total of 10–12 participants, but due to COVID-restrictions the pool of potential participants was limited. Due to the small sample size, it is possible that not all missing factors were identified. However, the amount of potentially missing factors is most likely negligible, since the overview was constructed from a meta-synthesis of qualitative literature in which a large sample of prosthesis users have presented factors of interest related to prosthesis use. These outcomes were all coded and combined in an overview, which was discussed during our focus group. Furthermore, it has been established that there is a wide range of factors and the importance of the various factors vary from person to person. Therefore it is possible that potentially missing factors were more personal factors, opposed to factors of general interest.

Since this study was solely based on qualitative literature, it is possible that certain factors that were missing and added during the focus group have been investigated in other types of studies. For example, most of the factors that were added based on the focus group can be found in quantitative studies. Especially the factors from the theme ‘walking’ have been quantified and studied using physical tests. Even though the results of the meta-synthesis were reviewed and complemented by the participants in the focus group, there is a possibility there are still factors missing that are only discussed in quantitative literature.

Lastly, as was shown in Table 2, six studies met all the CASP criteria. No studies were excluded based on the quality screening, but the quality of the studies may have had some impact on the outcome. The- two criteria most studies failed to meet were (1) description of the relationship between the researchers and participants and (2) description of the ethical consideration. A pre-existing relationship between researcher and participant could have had some influence the outcome of the study and with that on the validity. In four studies, it was mentioned that participants were recruited via clinicians who worked for companies the authors had no affiliation with [19,29,35,41]. This could indicate there was no pre-existing relationship. Furthermore, all studies focussed on individual prosthesis use and not on treatment provided by a clinician. In the latter case, prosthesis users might feel uncomfortable talking to their own clinician about negative experiences. Since this was not the case, we assume a potential pre-existing relationship did not affect the outcome of the included studies.

For the ethical consideration three studies did report approval from a local ethical committee or review board, but failed to mention the use of written informed consent or how participants were informed about the study [30,35,36]. Two studies did not report approval from a local ethical committee or review board, but did describe the use of written informed consent or how the participants were informed [29,41]. Even though these studies did not check all the boxes for this criterion on the quality assessment, some form of ethical consideration was shown. Therefore we do not think this had a big influence on the outcome of these studies.

## Conclusion

Our meta-synthesis and focus group results have provided an extensive overview of 94 factors that may influence the prosthesis use of lower-limb prosthesis users with a TFA or KD. The large number of factors demonstrates that there is a great variety between prosthesis users and the factors that influence their prosthesis use. Therefore, it is important to take individual preferences into account for the selection of a new prosthesis. The created overview can be used to improve the shared-decision making process between prosthesis users and clinicians to steer them towards the most ideal prosthesis for each individual user.

## Supporting information

S1 Table Consolidated criteria for reporting qualitative research (COREQ). (PDF)

S2 Table Preliminary, pre-final and final framework. (PDF)

S3 Table PRISMA 2020 checklist. (PDF)

S1 Text Search terms. (PDF)
