# Supplementary material for: User-relevant factors influencing the prosthesis use of persons with a transfemoral amputation or knee-disarticulation: A meta-synthesis of qualitative literature and focus group results
Source: PLoS One. 2023 Jan 17;18(1):e0276874. doi: 10.1371/journal.pone.0276874 (PMC9844830; doi:10.1371/journal.pone.0276874)
Supplement: S1 Table — (PDF) [file pone.0276874.s001.pdf]

S2 Table: Consolidated criteria for reporting qualitative research (COREQ)

No	Item	Study
**Domain 1: Research team and reflexivity**		
Personal Characteristics		
1	Interviewer/facilitator	Focus group was moderated by Sacha van Twillert (SvT), who was assisted by Charlotte Bosman (CB) and Esther van Veen (EvV).
2	Credentials	SvT: PhD, expert in implementation; CB: PhD candidate medical science; EvV: MSc-medical student.
3	Occupation	Researchers.
4	Gender	All female.
5	Experience and training	SvT has experience in moderating focus groups.
Relationship with participants		
6	Relationship established	Researchers and participants were not acquainted prior to the focus group .
7	Participant knowledge of the interviewer	At the start of the focus group, all three researchers were introduced. SvT as an experienced moderator, CB as main researcher for the project and EvV as student-assistant.
8	Interviewer characteristics	SvT was not biased and was invited as moderator based on her experience. CB is PhD candidate working on a larger project that also includes this focus group and who has created the pre-final framework.
**Domain 2: Study design**		
Theoretical framework		
9	Methodological orientation and theory	Framework approach.
10	Sampling	Purposive, based on eligibility criteria (Adults, amputation through or above the knee, at least one year experience with prosthesis). Selected and invited by an experienced prosthetist.

11	Method of approach	Eligible participants received an invitation and information letter via mail from their prosthetist.
12	Sample size	Eight were present at focus group, six were eligible (two did not meet inclusion criteria)
13	Non-participation	18 eligible participants were invited and ten responded. Two cancelled on the day of the focus group.
Setting		
14	Setting of data collection	Meeting room at the University Medical Center Groningen.
15	Presence of non-participants	SvT, CB and EvV.
16	Description of sample	See Table 3.
Data collection		
17	Interview guide	CB constructed guidelines with questions for the focus group, which were discussed with SvT. The guidelines consisted of two open questions, followed by the overview of factors.
18	Repeat interviews	N/A
19	Audio/visual recording	Audiorecorded.
20	Field notes	During the focus group, additional factors that were mentioned by participants were written down.
21	Duration	90 minutes.
22	Data saturation	The themes and factors from the pre-final framework that was formed during the meta-synthesis were discussed and supplemented during a focus group with lower limb prosthesis users.
23	Transcripts returned	After the transcription, EvV wrote a three-page summary of the focus group. The overview of themes and factors was added to this document and it was send to all participants via email. They were invited to respond with comments or corrections.
**Domain 3: Analysis and findings**		
Data analysis		
24	Number of data coders	Two reviewers.
25	Description of coding tree	The coding three consisted of 94 factors, divided over seven categories (see Fig. 2).
26	Derivation of themes	Most were identified in advance, except for the theme 'walking'.
27	Software	Atlas. Ti version 8.

28	Participant checking	All participants received a summary of the focus group and the overview of factors via email. One responded saying it was complete.
Reporting		
29	Quotations presented	Quotes can be found in the Results and are linked to participants. Participant demographics can be found in Table 3.
30	Data and findings consistent	Yes. Participants agreed with most factors that were identified in the literature review.
31	Clarity of major themes	See Results.
32	Clarity of minor themes	See Results.
