# Supplementary material for: User-relevant factors influencing the prosthesis use of persons with a transfemoral amputation or knee-disarticulation: A meta-synthesis of qualitative literature and focus group results
Source: PLoS One. 2023 Jan 17;18(1):e0276874. doi: 10.1371/journal.pone.0276874 (PMC9844830; doi:10.1371/journal.pone.0276874)
Supplement: S2 Table — (PDF) [file pone.0276874.s002.pdf]

S3 Table: Preliminary, pre-final and final framework

Theme	Factor	Definition	Framework		
			PL	PF	F
Prosthesis related factors	Easy to control	Ease of controlling the prosthesis	x	x	x
	Reliability	Whether the prosthesis does what you want, when you want	x	x	x
	Life-like appearance	Whether the prosthesis looks like a real leg	x		
	Size	The size of the prosthesis	x		
	Appearance in combination with clothing	Appearance with prosthesis under clothing (e.g. not being able to wear certain clothes and/or not wanting to wear certain clothes due to the prosthesis)	x	x	x
	Wearing comfort	How comfortable you can sit, stand and walk with the prosthesis	x	x	x
	Durability	Lifetime of the prosthesis and the separate parts of the prosthesis	x	x	x
	Donning/doffing	Effort needed to put the prosthesis on and off	x	x	x
	Noise	Noise the prosthesis makes during movements	x	x	x
	Weight	Weight of the prosthesis	x	x	x
	Heat/sweating	Heat and sweating while wearing the prosthesis	x	x	x
	Cold	Conduction of cold through the prosthesis	x	x	x
	Easy to clean	Ease of cleaning the prosthesis	x	x	
	Water/dirt proof	Resistance of prosthesis to water and dirt	x	x	x
	Self-maintenance	Maintenance of prosthesis that is done by the user themselves	x	x	x
	Functionality	Having a (clear) function	x	x	x
	Speed of movements	Speed of movements with prosthesis	x	x	
	Natural movements	Being able to make natural movements with the prosthesis	x	x	x

	Body balance	Having a more natural posture with the prosthesis, being more in balance/symmetric	x	x	
	Accessories	Accessories and tools that can be attached to a prosthesis or can be used to ease specific activities	x		
	Prosthesis fit	Fit of the prosthesis and changes therein, for example slipping of the prosthesis during activities or a poor prosthesis fit as a result of gaining/losing weight	x	x	x
	Usability	Ease of using a prosthesis	x	x	x
	Vulnerability/robustness	Ease in which the prosthesis can be damaged from the outside	x	x	x
	Safety	Whether or not the prosthesis use felt safe		x	
	Suspension	The method used to attach the prosthesis to the residual limb		x	x
	Prosthetic components	Components used for the prosthesis (e.g. foot, ankle, knee)		x	
	Alignment	The way the prosthetic components are aligned		x	
	Weightbearing capacity of stump	To what extend the stump can bear the bodyweight in the socket		x	
	Compensatory movements	Movements used to achieve functional motor skills when a normal movement pattern is unavailable		x	
	Appearance of prosthesis	How the prosthesis looks		x	x
	Shoe options	To what extent the use of the prosthesis affects the choice of shoe (height of heel, weight, type of sole)			x

**Activities and participation**	Performing family role	Fulfilling family functions and needs (e.g. spouse, parent, breadwinner)	x	x	x
	Leisure activities	Hobbies and sports	x	x	x
	Self-care	Taking care of yourself (e.g. taking a shower, getting dressed or using the restroom)	x	x	x
	Household	Performing household activities (e.g. hanging laundry, vacuuming, cooking)	x	x	x

	Transport	Moving yourself by car, bike or other transport methods.	x	x	x
	Work/school	Activities related to school and/or work.	x	x	x
	Multi-tasking	Performing multiple activities simultaneously	x	x	x
	Everyday activities / Daily living activities	Activities during the day (e.g. getting out of bed, eating, shopping etc.)		x	x
	Prioritizing activities	Having to give priority to certain activities because you are hampered by your prosthesis use (e.g. fatigue, pain or pressure spots)		x	x
	Activity level	How active a prosthesis user is		x	x

**Physical**	Gender	Gender of a person	x	x	x
	Origin of limb loss	Cause of limb loss, for example: congenital limb deficiency, trauma, malignancy, etc.	x	x	x
	Level of limb loss	Portion of a limb that is still present	x	x	x
	Phantom limb pain	Painful sensations of the missing limb, like it is still attached	x	x	x
	Phantom limb sensations	Non-painful sensations of the missing limb, like it is still attached	x		x
	Overuse symptoms	Pain symptoms due to overuse of for instance neck, back or unaffected leg. This also includes the prevention of these symptoms	x	x	x
	Skin irritation	Skin irritation, redness or inflammation of the stump skin due to prosthesis use	x	x	x
	Physical effort needed to control	Physical effort that is needed to control the prosthesis	x	x	
	Posture	Body posture when using the prosthesis			x
	Mobility	To what extend you are able to go wherever you want		x	x
	Pain	Experienced pain in the body that is not due to overuse (e.g. nerve pain)		x	x
	Physical abilities/restrictions	What is and is not physically possible as a result of the amputation and prosthesis use		x	x

	Condition sound limb	The condition of the sound limb, e.g. sensitivity, sensibility or overuse		x	
	Condition stump	The condition of the stump, e.g. sensitivity, sensibility or loss of strength		x	
	Stump sensibility	How sensitive the stump is to touch, movement and exertion			x
	General health	Whether or not a person has co-morbidities that interfere with prosthesis use		x	
**Social**	Support from family/friends	Supportive network with family and friends	x	x	x
	Performing social role	Fulfilling social functions and needs (e.g. as a friend, set of duties related to occupational status, in community), not related to the family	x	x	x
	Peer support	Support from other persons with lower limb deficiencies	x	x	x
	Reactions from public	Reactions in public about having a prosthesis/lower limb deficiency (e.g. staring, unwanted attention, questions)	x	x	x
	Fitting in	Belonging to a certain group; be the same as others	x	x	x
	Advertisement	The influence of promotion and advertisement about lower limb prostheses on the prosthesis use	x	x	x
	Anonymity	Not wanting to stand out or be stared at	x	x	x
	Giving support to family/friends	Take away the worries of family/friends	x	x	x
	Modelling	Having an exemplary function for others, for instance showing them how you deal with having a short leg	x		
	Prejudice	E.g. other people taking over your tasks because they think you cannot do them yourself, while in reality that does not have to be the case	x	x	x
	Pressure of others	Pressure that people exert to wear or not to wear a (particular) prosthesis	x	x	x
	Help (giving/receiving)	Asking for and receiving help as a prosthesis user, as well as being able to help others as a prosthesis user		x	

	Social interactions	The way people interact, for example, body language, shaking hands, having conversations and establishing new relationships and whether this is affected by prosthesis use or wheelchair use (e.g. not being able to talk at the same eye level from a wheelchair)		x	x
	Reactions from friends/family	Positive or negative reactions from your family or friends about prosthesis			x

**Rehabilitation, costs and prosthetist services**	Access to service	Having access to rehabilitation services (e.g. travel distance, physical access, making appointments, acceptability of services)	x	x	x
	Cost of prosthesis	Costs of purchasing prosthesis and reimbursements for this	x	x	x
	Cost of maintenance	Costs of maintenance prosthesis and reimbursements for this	x	x	x
	Information services	Being informed about the possible prosthesis options with their pros and cons	x	x	x
	Prosthesis training	Efficacy and satisfaction of prosthesis training services	x	x	
	Time investment	Time-investment for choosing and fitting a prosthesis, prosthesis training, maintenance, follow-up and travel time	x	x	x
	Professional prosthesis maintenance	Prosthesis maintenance that should be done by a professional (like the prosthetist or therapist)	x	x	
	Expertise of guidance	Expertise of the professionals involved in the process of choosing a prosthesis	x	x	x
	Own costs	Own costs (deductible) of prosthesis, maintenance and training	x	x	x
	Procedure insurances	Procedure and needed 'paperwork' that is required to request reimbursements from the health insurer	x	x	x
	Trial period	Short period in which someone can try out one or more prostheses at home, before actually choosing a prosthesis	x	x	x

	Availability new developments	Time that it takes until a certain new development in hand prosthesis is commercially available	x	x	
	Rehabilitation program	The entire rehabilitation trajectory from initial amputation to receiving the prosthesis (including guidance and training)		x	x
	Adjustment to daily life	Adjustments that had to be made within the home and at work (e.g. adjustments to the car)		x	
	Communication with caregivers	This includes all members of the rehabilitation team (rehabilitation doctor, physiotherapist, occupational therapist, psychologist, prosthetist etc.)		x	x
	Relationship with prosthetist	To what extend the good or bad relationship with the prosthetist had on the prosthesis use		x	x
	User involvement	To what extend the prosthesis user is involved in the rehabilitation program and prosthesis choice		x	x
**Mental**	Motivation	Motivation for prosthesis use	x	x	
	Attitude	Personal factors, character traits and principles that influences how someone thinks or acts in certain situations	x	x	x
	Coping	Way someone deals with difficult situations (e.g. problem solving, handling reactions of others, etc.)	x	x	x
	Expectations	Someone's expectations in regard of prosthesis use and whether these expectations are realistic	x	x	x
	Self-confidence	Self-confidence in regard to wearing a prosthesis	x	x	x
	Time since limb loss	Time in years since limb loss	x	x	
	Feeling disabled	Feeling that you are disabled with/without prosthesis	x	x	x
	Independence	Feeling that you are independent with/without prosthesis	x	x	x
	Body embarrassment	Embarrassment for the own body with/without prosthesis	x	x	x
	Need for prosthesis	Need for a prosthesis/ experiencing the added value of a prosthesis	x	x	x
	Self-image	How someone looks at themselves in regard to abilities, appearance and personality	x	x	x

	Autonomy	Freedom of someone to decide for which activities he/she want to use the prosthesis (including unintended use) and how to use it	x	x	
	Embodiment	Feeling that the prosthesis is part of the body/ feeling ‘complete’ with the prosthesis	x	x	x
	Mental effort needed to control	Mental effort that is needed to control the prosthesis	x	x	x
	Curiosity	Having a desire to know how it would be to have a (particular) prosthesis	x		
	Acceptance	To what extend the prosthesis user has accepted the prosthesis and living with an amputation		x	x
	Reaction to amputation	How the prosthesis user felt about the amputation		x	
	Reaction to first prosthesis	How the prosthesis user felt about their first prosthesis and the influence it has on current prosthesis use		x	
	Relying on prosthesis	To what extent the user has to rely on the prosthesis day-to-day		x	x
	Feeling inferior	Feeling less than persons without an amputation		x	
	Feeling depressed	Feeling depressed due to the amputation or prosthesis and use of a prosthesis		x	
	Goals	Which goals and expectations the prosthesis user has		x	
	Fear of future health problems	E.g. overuse complaints, back and neck pain or issues with the intact limb		x	x
	Emotions	Positive and negative emotions cause by the prosthesis (e.g. feeling down or being proud of their prosthesis)			x

**Walking**	Walking confidence	Feeling of confidence when walking with the prosthesis		x	x
	Walking speed	Speed of running and the possibility to vary it		x	x
	Use of (walking) aids	The use of aids e.g. crutches or walker		x	x
	Fear of falling	Fear of falling with or without the prosthesis		x	x
	Surface	The influence of walking surface on gait		x	x
	Balance	Ability to maintain balance during walking		x	x

	Running	Being able to run			x
	Walking backwards	Being able to walk backwards			x
	Steps	Going up and down small steps (e.g. curbs)			x
	Walking distance	How far you can walk with the prosthesis			x

PL, preliminary framework; PF, pre-final framework; F, Final framework

The preliminary framework was taken from Kerver et al. [21] and used to code the included studies in the meta-synthesis. During/after the meta-synthesis certain factors were added or deleted, which formed the pre-final framework. The pre-final framework was discussed during the focus group. After the results of the focus group were processed, we formed the final framework.
