# Supplementary material for: User-relevant factors influencing the prosthesis use of persons with a transfemoral amputation or knee-disarticulation: A meta-synthesis of qualitative literature and focus group results
Source: PLoS One. 2023 Jan 17;18(1):e0276874. doi: 10.1371/journal.pone.0276874 (PMC9844830; doi:10.1371/journal.pone.0276874)
Supplement: S1 Text — (PDF) [file pone.0276874.s004.pdf]

S1 text: Search terms

PubMed

(“lower extremity”[Mesh] OR lower extremit* [tiab] OR Lower limb* [tiab] OR leg [tiab] OR legs [tiab] OR transfemor* [tiab] OR knee-disartic* [tiab] OR knee-exartic*[tiab] OR above-knee [tiab] OR above the knee [tiab] OR through the knee [tiab] OR LLA [tiab])

AND

("Artificial Limbs"[Mesh] OR artificial limb* [tiab] OR micro-processor knee*[tiab] OR microprocessor knee*[tiab] OR MPK [tiab] OR NMPK [tiab] OR AAK [tiab] OR conventional knee*[tiab] OR CMK [tiab] OR mechanical knee*[tiab] OR C-leg[tiab] OR genium [tiab] OR rheo[tiab] OR computer-controlled knee*[tiab] OR kenevo [tiab] OR electronic knee*[tiab] OR autoadaptive knee*[tiab] OR auto-adaptive knee*[tiab] OR LLP [tiab])

AND

((“qualitative Research” [Mesh] OR “Focus group” [Mesh] OR “Focus group*” [tiab] OR questionnaire* [tiab] OR survey* [tiab] OR interview* [tiab] OR opinion* [tiab] OR experien* [tiab] OR view* [tiab] OR concern* [tiab] OR motivat* [tiab] OR value* [tiab] OR desir* [tiab] OR perspective* [tiab] OR wish* [tiab] OR expectat* [tiab] OR attitud* [tiab] OR feel* [tiab] OR “patient satisfaction” [Mesh] OR useful* [tiab] OR satisf* [tiab] OR functional* [tiab] OR usage* [tiab] OR wear* [tiab] OR advantage* [tiab] OR disadvantage* [tiab] OR embodiment* [tiab] OR “rejection rat*” [tiab] OR cosmetic* [tiab] OR esthetic* [tiab] OR aesthetic* [tiab] OR utilit* [tiab] OR comfort* [tiab] OR requirement* [tiab] OR need* [tiab] OR “quality of life” [Mesh] OR quality of life* [tiab] OR “Cost-benefit analysis” [Mesh] OR Accept* [tiab] OR reject* [tiab] OR “value based health care” [tiab] OR effectiv* [tiab] OR efficien* [tiab] OR prefer* [tiab] OR econom* [tiab] OR costefficien* [tiab] OR cost-efficien* [tiab] OR opus [tiab] OR tapes* [tiab] OR quest* [tiab] OR qualitativ* [tiab] OR narrativ* [tiab] OR mixed method* [tiab] OR case stud* [tiab] OR PEQ [tiab] OR “plus-m” [tiab] OR QES [tiab] OR “thematic analys*” [tiab] OR “qualitative stud*” [tiab] OR “qualitative analys*” [tiab] OR “qualitative research*” [tiab] OR qualitative method*[tiab]) NOT (transplant* [tiab] OR pediatr* [tiab] OR 3d* [tiab]))

Psycinfo

(TI (lower extremit* OR Lower limb* OR leg OR legs OR transfemor* OR knee-disartic* OR knee-exartic* OR above-knee OR above the knee OR through the knee OR LLA) OR AB (lower extremit* OR Lower limb* OR leg OR legs OR transfemor* OR knee-disartic* OR knee-exartic* OR above-knee OR above the knee OR through the knee OR LLA))

AND

(DE "Prostheses" OR TI (artificial limb* OR micro-processor knee* OR microprocessor knee* OR MPK OR NMPK OR AAK OR conventional knee* OR CMK OR mechanical knee* OR C-leg OR genium OR rheo OR computer-controlled knee* OR kenevo OR electronic knee* OR autoadaptive knee* OR auto-adaptive knee* OR LLP) OR AB (artificial limb* OR micro-processor knee* OR microprocessor knee* OR MPK OR NMPK OR AAK OR conventional knee* OR CMK OR mechanical knee* OR C-leg OR genium OR rheo OR computer-controlled knee* OR kenevo OR electronic knee* OR
autoadaptive knee* OR auto-adaptive knee* OR LLP))

AND

DE "Qualitative Methods" OR DE "Focus Group" OR DE "Grounded Theory" OR DE "Interpretative Phenomenological Analysis" OR DE "Narrative Analysis" OR DE "Semi-Structured Interview" OR DE "Thematic Analysis" OR DE "Mixed Methods Research" OR DE "Quality of Life" OR DE "Health Related Quality of Life" OR DE "Quality of Work Life" OR DE "Costs and Cost Analysis" OR DE "Budgets" OR DE "Cost Containment" OR DE "Health Care Costs" OR DE "Money" OR DE "Client Satisfaction" OR TI (Focus group* OR questionnaire* OR survey* OR interview* OR opinion* OR experien* OR view* OR concern* OR motivat* OR value* OR desir* OR perspective* OR wish* OR expectat* OR attitud* OR feel* OR useful* OR satisf* OR functional* OR usage* OR wear* OR advantage* OR disadvantage* OR embodiment* OR rejection rat* OR cosmetic* OR esthetic* OR aesthetic* OR utilit* OR comfort* OR requirement* OR need* OR quality of life* OR Accept* OR reject* OR value based health care OR effectiv* OR efficien* OR prefer* OR econom* OR costefficien* OR cost-efficien* OR opus OR tapes* OR
quest* OR qualitativ* OR narrativ* OR mixed method* OR case stud* OR PEQ OR “plus-m” OR QES OR “thematic analys*” OR qualitative stud* OR qualitative analys* OR qualitative research* OR qualitative method*) OR AB (Focus group* OR questionnaire* OR

survey* OR interview* OR opinion* OR experien* OR view* OR concern* OR motivat* OR value* OR desir* OR perspective* OR wish* OR expectat* OR attitud* OR feel* OR useful* OR satisf* OR functional* OR usage* OR wear* OR advantage* OR disadvantage* OR embodiment* OR rejection rat* OR cosmetic* OR esthetic* OR aesthetic* OR utilit* OR comfort* OR requirement* OR need* OR quality of life* OR Accept* OR reject* OR value based health care OR effectiv* OR efficien* OR prefer* OR econom* OR costefficien* OR cost-efficien* OR opus OR tapes* OR quest* OR qualitativ* OR narrativ* OR mixed method* OR case stud* OR PEQ OR "plus-m" OR QES OR "thematic analys*" OR qualitative stud* OR qualitative analys* OR qualitative research* OR qualitative method*)

Embase

('lower limb'/exp OR ('lower extremit*' OR 'Lower limb*' OR leg OR legs OR transfemor* OR 'knee-disartic*' OR 'knee-exartic*' OR 'above-knee' OR ‘above the knee’ OR ‘through the knee’ OR LLA):ab,ti)

AND

('electric limb prosthesis'/exp OR 'leg prosthesis'/exp OR ('artificial limb*' OR 'micro-processor knee*' OR 'microprocessor knee*' OR MPK OR NMPK OR AAK OR 'conventional knee*' OR CMK OR 'mechanical knee*' OR 'C-leg' OR genium OR rheo OR 'computer-controlled knee*' OR kenevo OR 'electronic knee*' OR 'autoadaptive knee*' OR 'auto-adaptive knee*' OR LLP):ab,ti)

AND

('qualitative research'/exp OR 'focus group'/exp OR 'patient satisfaction'/exp OR 'cost benefit analysis'/exp OR ('Focus group*' OR questionnaire* OR survey* OR interview* OR opinion* OR experien* OR view* OR concern* OR motivat* OR value* OR desir* OR perspective* OR wish* OR expectat* OR attitud* OR feel* OR useful* OR satisf* OR functional* OR usage* OR wear* OR advantage* OR disadvantage* OR embodiment* OR 'rejection rat*' OR cosmetic* OR esthetic* OR aesthetic* OR utilit* OR comfort* OR requirement* OR need* OR 'quality of life*' OR Accept* OR reject* OR 'value based health care' OR effectiv* OR efficien* OR prefer* OR econom* OR costefficien* OR cost-efficien* OR opus OR tapes* OR quest* OR qualitativ* OR narrativ* OR 'mixed method*' OR 'case stud*' OR PEQ OR 'plus-m' OR QES OR 'thematic analys*' OR 'qualitative stud*' OR 'qualitative analys*' OR 'qualitative research*' OR 'qualitative method*'):ab,ti NOT (transplant*:ab,ti OR pediatr*:ab,ti OR 3d*:ab,ti))

CINAHL

(TI (lower extremit* OR Lower limb* OR leg OR legs OR transfemor* OR knee-disartic* OR knee-exartic* OR above-knee OR ''above the knee'' OR LLA) OR AB (lower extremit* OR Lower limb* OR leg OR legs OR transfemor* OR knee-disartic* OR knee-exartic* OR above-knee OR ''above the knee'' OR LLA))

AND

((MH "Orthopedic Prosthesis+") OR TI (artificial limb* OR micro-processor knee* OR microprocessor knee* OR MPK OR NMPK OR AAK OR conventional knee* OR CMK OR mechanical knee* OR C-leg OR genium OR rheo OR computer-controlled knee* OR kenevo OR electronic knee* OR autoadaptive knee* OR auto-adaptive knee* OR LLP) OR AB (artificial limb* OR micro-processor knee* OR microprocessor knee* OR MPK OR NMPK OR AAK OR conventional knee* OR CMK OR mechanical knee* OR C-leg OR genium OR rheo OR computer-controlled knee* OR kenevo OR electronic knee* OR autoadaptive knee* OR auto-adaptive knee* OR LLP))

AND

((MH "Focus Groups") OR (MH "Qualitative Studies+") OR (MH "Cost Benefit Analysis") OR (MH "Patient Satisfaction+") OR (MH "Quality of Life+") OR TI (Focus group* OR questionnaire* OR survey* OR interview* OR opinion* OR experien* OR view* OR concern* OR motivat* OR value* OR desir* OR perspective* OR wish* OR expectat* OR attitud* OR feel* OR useful* OR satisf* OR functional* OR usage* OR wear* OR advantage* OR disadvantage* OR embodiment* OR rejection rat* OR cosmetic* OR esthetic* OR aesthetic* OR utilit* OR comfort* OR requirement* OR need* OR quality of life* OR Accept* OR reject* OR value based health care OR effectiv* OR efficien* OR preferenc* OR econom* OR costefficien* OR cost-efficien* OR opus OR tapes* OR quest* OR qualitativ* OR narrativ* OR mixed method* OR "case stud*" OR PEQ OR "plus-m" OR QES OR "thematic analys*" OR qualitative stud* OR qualitative analys* OR qualitative research* OR qualitative method*) OR AB (Focus group* OR questionnaire* OR survey* OR interview* OR opinion* OR experien* OR view* OR concern* OR motivat* OR value* OR desir* OR perspective* OR wish* OR expectat* OR attitud* OR feel* OR useful* OR satisf* OR functional* OR usage* OR wear* OR advantage* OR disadvantage* OR embodiment* OR rejection rat* OR cosmetic* OR esthetic* OR

aesthetic* OR utilit* OR comfort* OR requirement* OR need* OR quality of life* OR Accept* OR reject* OR value based health care OR effectiv* OR efficien* OR preferenc* OR econom* OR costefficien* OR cost-efficien* OR opus OR tapes* OR quest* OR qualitativ* OR narrativ* OR mixed method* OR “case stud*” OR PEQ OR “plus-m” OR QES OR “thematic analys*” OR qualitative stud* OR qualitative analys* OR qualitative research* OR qualitative method*)) NOT (TI (transplant* OR pediatr* OR 3d*) OR AB (transplant* OR pediatr* OR 3d*))

Web of Science

(“lower extremit*” OR “Lower limb*” OR leg OR legs OR transfemor* OR “knee-disartic*” OR “knee-exartic*” OR “above-knee” OR ′′above the knee′′ OR LLA)

AND

(“artificial limb*” OR “micro-processor knee”* OR “microprocessor knee*” OR MPK OR NMPK OR AAK OR “conventional knee*” OR CMK OR “mechanical knee*” OR “C-leg” OR genium OR rheo OR “computer-controlled knee*” OR kenevo OR “electronic knee*” OR “autoadaptive knee*” OR “auto-adaptive knee*” OR OR LLP)

AND

(“Focus group*” OR questionnaire* OR survey* OR interview* OR opinion* OR experien* OR view* OR concern* OR motivat* OR value* OR desir* OR perspective* OR wish* OR expectat* OR attitud* OR feel* OR useful* OR satisf* OR functional* OR usage* OR wear* OR advantage* OR disadvantage* OR embodiment* OR rejection rat* OR cosmetic* OR esthetic* OR aesthetic* OR utilit* OR comfort* OR requirement* OR need* OR quality of life* OR Accept* OR reject* OR “value based health care” OR effectiv* OR efficien* OR preferenc* OR econom* OR costefficien* OR “cost-efficien*” OR opus OR tapes* OR quest* OR qualitativ* OR narrativ* OR “mixed method*” OR “case stud*” OR PEQ OR “plus-m” OR QES OR “thematic analys*” OR “qualitative stud*” OR “qualitative analys*” OR “qualitative research*” OR “qualitative method*”)
